# Supplementary material for: Comparative study on abortion characteristics of Nsa CMS and Pol CMS and analysis of long non-coding RNAs related to pollen abortion in Brassica napus
Source: PLoS One. 2023 Apr 13;18(4):e0284287. doi: 10.1371/journal.pone.0284287 (PMC10101420; doi:10.1371/journal.pone.0284287)
Supplement: S1 File — (DOCX) [file pone.0284287.s008.docx]

**
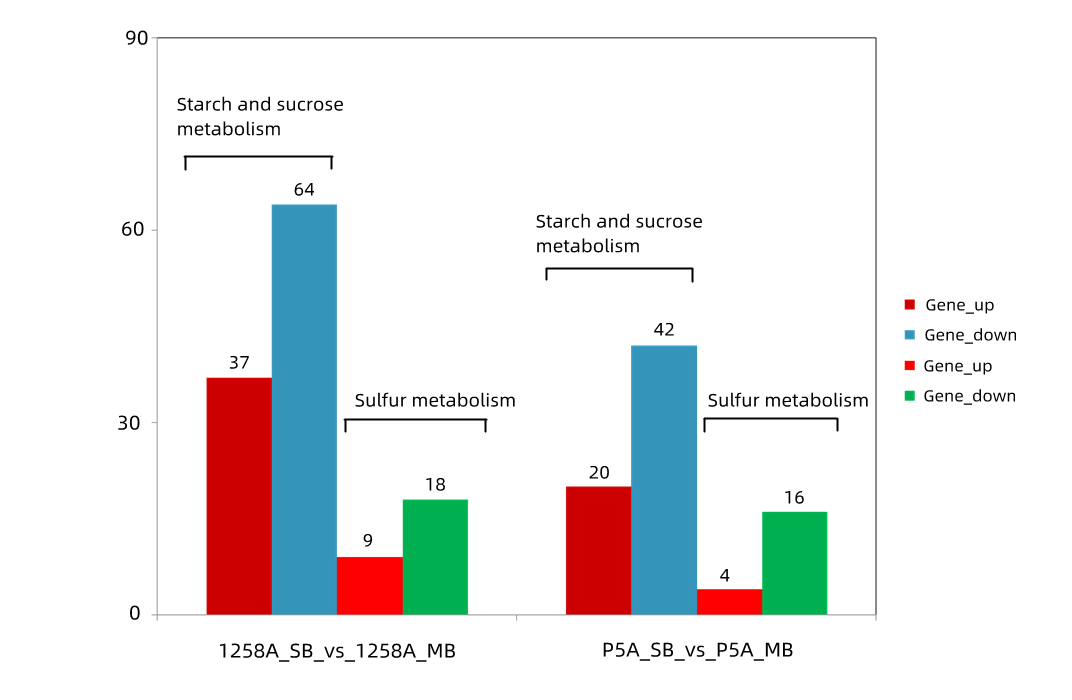
**

**Supplementary Figure 1** Statistical analysis of differentially expressed genes in Starch and sucrose metabolism and Sulfur metabolismStarch and sucrose metabolism in 1258A SB, 1258B MB, P5A SB and P5A MB buds.

**Supplementary Table 1 Primers used for RT-qPCR.**

| Primer name | Primer sequence |
| --- | --- |
| LOC106431114-qPCR-F | ATGCTACTATGAGTGGGC |
| LOC106431114-qPCR-R | AGTTTCTGGTCGTGTTCT |
| LOC106440633-qPCR-F | ATCGTCACCTCCACTTGT |
| LOC106440633-qPCR-R | TACCTTGCCCTTCTGTTC |
| LOC106442156-qPCR-F | GTCTGAACTTCGCAAAAC |
| LOC106442156-qPCR-R | ACCAGCAAGCATCTCTCT |
| LOC106419631-qPCR-F | CGAGAGAAAGTGGTGGTT |
| LOC106419631-qPCR-R | GTAGACTGTGACGGAAGC |
| LOC106353728-qPCR-F | TGGCTCATATTAGTGGGT |
| LOC106353728-qPCR-R | TGCGTTTATTCTGTCTTC |
| LOC106360908-qPCR-F | TTACGCCTTATACCCACC |
| LOC106360908-qPCR-R | TGCCATTGCTATCACCTT |
| MERGE.48025.1-qPCR-F | TAGCCAGGCAGATTTGTA |
| MERGE.48025.1-qPCR-R | ATGTGTGACTGTGGGACC |
| MERGE.32337.5-qPCR-F | GTGCTCCACCGATTGACT |
| MERGE.32337.5-qPCR-R | CTTTGGCTGCTCCTACGA |
| MERGE.79317.2-qPCR-F | TCCGACAAAGCAGCGAAA |
| MERGE.79317.2-qPCR-R | CGAGGGCACTCAAAGCAA |
| MERGE.1241.3 -qPCR-F | TTACTACCAGACAACCGT |
| MERGE.1241.3 -qPCR-R | ATACCACAAGAACCAACC |
| MERGE.85690.1-qPCR-F | GACAACCAAAGATGAGGG |
| MERGE.85690.1-qPCR-R | GAGATCGGTGGAGACAAG |
| MERGE.11357.2-qPCR-F | CCGTCTCATTCGCACTGG |
| MERGE.11357.2-qPCR-R | CTAAATCGTCTCTCCCCT |


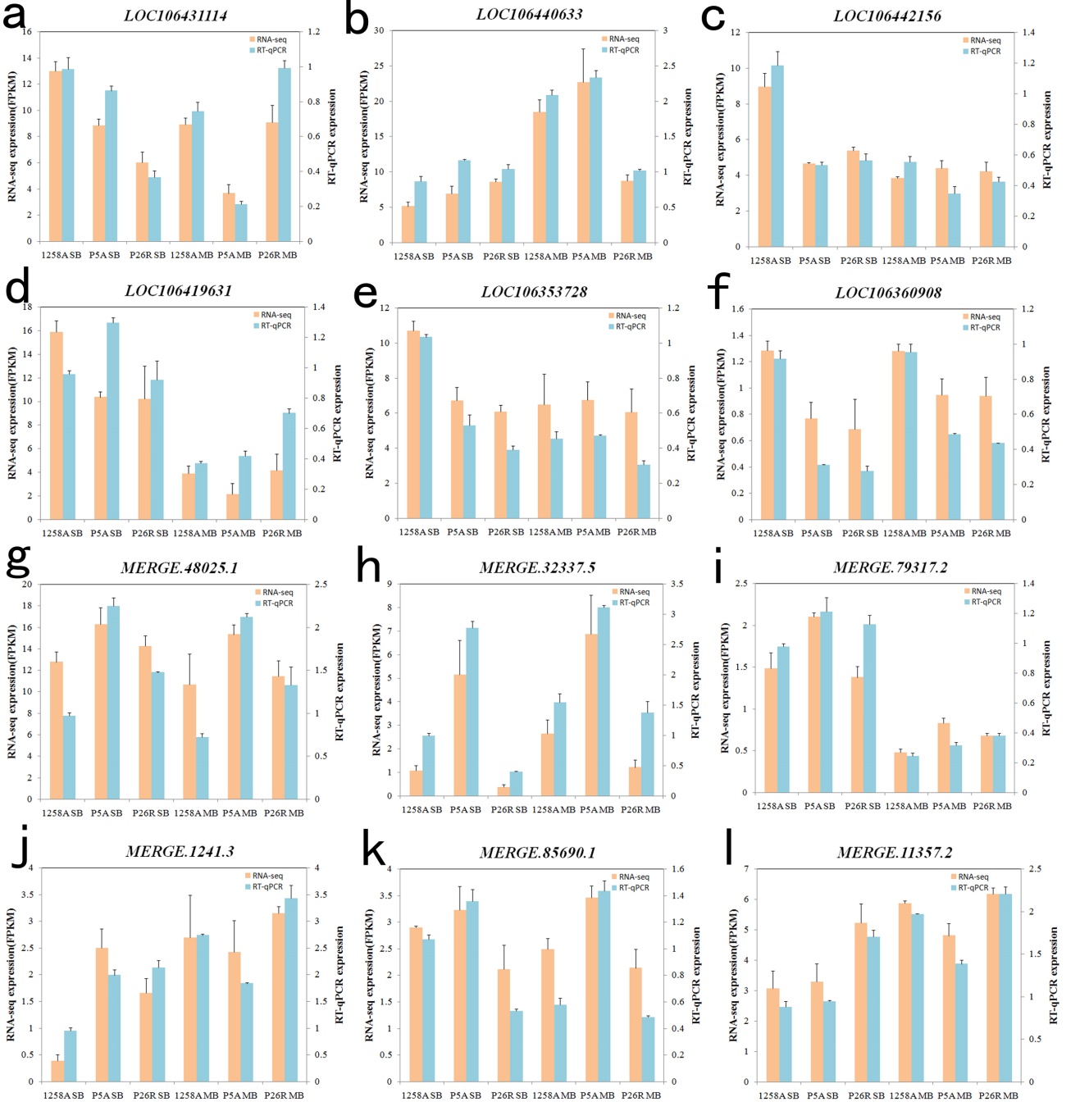


**Supplementary Figure 2 Real-time quantitive PCR(RT-qPCR) validation of selected genes from RNA sequencing.(a–f)**RT-qPCR results of selected differentially expressed genes. **(g–i)**RT-qPCR results of selected differentially expressed lncRNAs.
