# Supplementary material for: Comparative study on abortion characteristics of Nsa CMS and Pol CMS and analysis of long non-coding RNAs related to pollen abortion in Brassica napus
Source: PLoS One. 2023 Apr 13;18(4):e0284287. doi: 10.1371/journal.pone.0284287 (PMC10101420; doi:10.1371/journal.pone.0284287)
Supplement: Attachment — Submitted filename: Response to Reviewers.docx [file pone.0284287.s009.docx]

**Response to Reviewer1：**

Thank you very much for your comments！

1. First, we made a complete revision of the English grammar and structure in the manuscript.

2. We condensed the abstract and briefly summarized the results.

3. We mean that pollen can be produced normally in the anthers of the maintainer line (1258B) and not in the sterile line (1258A).

4. We added the biological repetition of the methods section and the differential analysis method in Figure 3.

5. We removed the "KEGG pathway" section in Figures 7 and Figures 8.

You may review our revised manuscript, thank you very much!

**Response to Reviewer2：**

Thank you very much for your comments！

1. Method part:

（1）We have added information on plant materials（P5A and P56B） according to your suggestion.

（2）We improved transcriptome sequencing to add biological duplicates. We added the method of determining physiological and biochemical indexes in anthers and improved the method information.

2. Results part:

（1）We rewrote the“Morphological and cytological characteristics of *Nsa* CMS and *Pol* CMS” part.The results of qRT-PCR are shown in the attached figure s2.

（2）We added the full name of the word "DE" and the reference to the "Fastp" section.

Thank you very much for your suggestion. We have revised the manuscript for your review.
